# Homeownership Matters: Impact of Homeownership on the Prevalence of Chronic Health Conditions in the United States

**DOI:** 10.5888/pcd21.230324

**Published:** 2024-05-16

**Authors:** Shams Rahman, David Steeb

**Affiliations:** 1 College of Global Population Health, University of Health Sciences and Pharmacy in St. Louis, Missouri

## Abstract

**Introduction:**

Homeownership is crucial for stability and healthy life. We examined the role of homeownership in predicting the prevalence of common chronic health conditions in the United States.

**Methods:**

We used 2020 Behavioral Risk Factor Surveillance System data (N = 401,958) to assess the association between homeownership and self-reported diagnosed diabetes, asthma, cancer, coronary heart disease (CHD), stroke, and kidney disease. We analyzed data by using logistic regression, adjusting for age, sex, race and ethnicity, education, employment, and income and computed odds ratios (ORs) and corresponding 95% CIs.

**Results:**

Most survey participants (66.8%) owned their residences. Age, marital status, education, and income significantly influenced homeownership. Odds of homeownership progressively increased with age, reaching a peak at 17.45 (95% CI, 16.21–18.79) for adults aged 65 years or older, and non-Hispanic White adults had the highest odds (OR = 3.34; 95% CI, 3.18–3.52). Compared with renters, homeowners generally had lower prevalence of chronic health conditions, especially among those aged 45 to 64 years. After adjusting for age, sex, and race and ethnicity, the odds of having chronic health conditions among renters were higher than those of homeowners: CHD, 1.39 (1.27–1.52); diabetes, 1.27 (1.20–1.35); asthma, 1.29 (1.23–1.36); stroke, 1.89 (1.71–2.09); and kidney disease, 1.59 (1.44–1.77).

**Conclusion:**

Homeownership can be used to predict the prevalence of several chronic health conditions. Considering its significant influence, public health initiatives should focus on housing-related interventions to improve population health.

**Summary**

**What is known on this topic?**

Stable housing significantly influences the prevalence of chronic health conditions and overall well-being; affordable homeownership is crucial for stable residence. Recognizing the distinction between “housing” and “homeownership” is important for shaping effective policies that prioritize sustainable homeownership over mere housing.

**What is added by this report?**

This study provides substantial evidence that homeownership is associated with a lower prevalence of chronic health conditions, acting as a robust protective factor across various age groups and serving as a reliable socioeconomic variable.

**What are the implications for public health practice?**

Promoting affordable homeownership could be a pivotal public health strategy, ensuring stability and potentially reducing the burden of chronic health conditions in the US.

## Introduction

The Universal Declaration of Human Rights recognizes “adequate housing” as a basic human right (1). Housing is among the most important social determinants of health (SDOH) (2,3). Homeownership is an essential pathway to economic security, social mobility, stability, generational wealth, and healthy life. Financial assets such as owning a house or land passed down from one generation to the next within families is referred to as generational wealth (4). Recent research conducted by the National Realtor Association reported that, on average, a homeowner gained $139,134 in equity in the last 5 years, and $218,505 in the last 10 years (5). Homeownership is also key to narrowing racial inequity gaps in income and wealth (6). Wealth and income are strongly associated with increased illness and death rates and reduced life expectancy (7). Poor health also leads to reduced and inadequate income, creating a health–poverty trap (7).

The relationship between adequate housing and health has been well established. Housing can affect health through multiple mechanisms. For example, homeowners have a stable place to live, and residential stability leads to improved health (8,9). In the US, most services — such as access to health care, schools, and financial, social, and municipal services — are linked to residential status and residential history (10). These SDOH also affect health and health outcomes. Owning a residence also contributes to better internal house conditions and affordability, which can impact health status (9,11).

Extensive research in recent decades has solidified the association between housing and health outcomes, prompting a paradigm shift that considers housing as an integral component of overall health (12,13). Advancing beyond age-old hypotheses to explore a new domain, the relationship between homeownership and health outcomes is necessary for contemporary public health. Homeownership, symbolizing stable and sustainable housing, offers a unique lens to enhance our understanding of community health.

Understanding the connection between homeownership and chronic health conditions is imperative in the field of public health. Recent decades have underscored the critical role of housing in overall health outcomes, yet a substantial knowledge gap exists concerning how homeownership, as a distinct housing aspect, influences chronic health conditions. This understanding holds vital implications for several reasons. Chronic health conditions are the leading cause of illness and death, responsible for 90% of the $4.1 trillion in annual health care expenditures in the US, necessitating their prioritization in public health efforts (14). Furthermore, homeownership extends beyond a mere housing arrangement, encompassing stability, community ties, social networks, and financial security — factors that can affect the risk and management of chronic health conditions. By exploring this under-researched area, we can identify interventions and policies to leverage homeownership to mitigate chronic health issues and enhance overall well-being. This study aims to establish homeownership as a critical determinant of health and social well-being, particularly in predicting the prevalence of major chronic health conditions in the US. By addressing this knowledge gap, we highlight the complex relationship between homeownership and chronic health conditions, enhancing our holistic approach to public health and housing policies. Our objective was to investigate the effect of residential status on the prevalence of key chronic health conditions in the US.

## Methods

We analyzed 2020 data from the Behavioral Risk Factor Surveillance System (BRFSS; N = 401,958). Details of the BRFSS cross-sectional survey, methods, sampling, data collection, and weights applied to calculate population estimates can be found at www.cdc.gov/brfss/index.html. Briefly, BRFSS is the largest telephone survey in the US, collecting self-reported prevalence data on chronic health conditions, risk behaviors, and preventive services use from a representative sample of adults aged 18 years or older. Primarily, the survey uses a stratified random sampling design. BRFSS data use 2 types of weights that account for the survey design and population characteristics, and they are calculated on the basis of each geographic stratum, number of telephones within sampled household, and number of adults aged 18 years or older living in the residence. The survey uses iterative proportional fitting (ie, raking) to adjust estimates for demographic differences between the sample and reference populations. Within the scope of this article and based on data availability, we examined the 6 leading chronic health conditions in the US with the highest disease burden and economic impact. Outcome variables were self-reported ever diabetes, asthma, cancer (other than skin), angina/coronary heart disease (CHD), stroke, and kidney disease. The exposure variable was homeownership.

We defined and used analytic variables based on the 2020 BRFSS questionnaires. The homeownership variable, derived from Core Section 8, queried respondents on owning or renting their homes; response options were “own,” “rent,” “other arrangements,” and “don’t know/not sure/refused.” For analytical precision, the “other arrangements” category was combined with “rent” due to similar housing statuses. Responses in the “don't know/not sure/refused” category for both homeownership and chronic health conditions were treated as missing values in all analyses. Chronic health conditions, obtained from Core Section 6, used a general question format: “Has a doctor, nurse, or other health professional ever told you had [condition]?” The conditions were angina or CHD, diabetes, stroke, cancer other than skin, asthma, and kidney disease. Response options for chronic health conditions, excluding “ever diabetes,” were yes, no, don't know/not sure/refused. For “ever diabetes,” response options were yes, “prediabetic or borderline,” “yes, during pregnancy,” no, and don't know/not sure/refused. To streamline analysis and ensure comparability, yes, “prediabetic or borderline,” and “yes, during pregnancy” were combined based on the assumption of their shared indication of altered glucose metabolism and similar risk profiles.

All analyses were performed in SAS 9.4 (SAS Institute, Inc). According to the BRFSS analysis manual, we calculated weighted estimates (population proportions and odds ratios [ORs]) by supplying appropriate strata, cluster, and weight information. SAS has specialized procedures for sample survey data that could incorporate design factors and the understanding of population characteristics. For descriptive statistics and sample distribution by sociodemographic characteristics we used PROC SURVEYFREQ. This procedure yields n-way frequency and cross tabulation tables for population totals and population proportions. We used PROC SURVEYLOGISTIC to construct our logistic regression models. PROC SURVEYLOGISTIC integrates complex survey sample designs, stratification, clustering, and unequal weighting and offers fitting a broad class of binary response models in the general form of g (π) = α + xβ. The associations between homeownership and demographic characteristics were also examined by using logistic regression. To illustrate the influence of the study design on this association, both unweighted and weighted ORs were computed.

We used a nested model approach to construct our adjusted logistic regression models. Initially, we began with just 2 demographic variables and systematically incorporated additional variables. We employed a sequential adjustment, progressing from age and sex in Model 1 to various sociodemographic variables in Model 5, enabling a nuanced exploration of how diverse demographic factors collectively influence homeownership likelihood. The use of multiple models enhanced the thorough exploration of the interplay between demographic factors and homeownership. This iterative process resulted in our final adjusted model, which encompassed a comprehensive set of covariates: sex, age, race and ethnicity, education, income, marital status, and employment. To evaluate the effect of these variable additions on model fit, we leveraged the likelihood ratio test, a conventional statistical technique for assessing the significance of model improvements within nested models. For adjusted models, certain categories of marital status, education, and employment were collapsed and combined to simplify analysis while preserving relevant characteristics within each group. In regression analyses the choice of referent groups aimed to consistently present ORs above 1, emphasizing increased odds for an easy and intuitive interpretation of study results. We present findings from descriptive and regression analyses and report population proportions, population-level ORs, and corresponding 95% CIs.

## Results

Most of the sample population (66.8%) reported living in their own residences, 26.9% were renting, and the remaining 6.3% were residing under “other arrangements” (Table 1). Individuals aged 65 years or older accounted for 21.9% of the sample, the largest proportion. The population was roughly equal between female (51.3%) and male (48.7%) respondents. The study population was predominantly non-Hispanic White (61.8%), followed by Hispanic (17.8%), Black (11.8%), and Asian (5.6%). Most of the study population was married (50.3%) or had never married (24.8%). The largest proportion of the study population attended some college or graduated from college or technical school (59.8%), followed by high school graduates (27.8%) and those who did not complete high school (12.5%). The largest proportion of respondents in the employment category were those employed for wages (46.8%), followed by retired people (19.6%). The largest proportion of respondents in the income category were those who earned $50,000 or more (53.3%), and the remaining proportions varied. 

**Table 1:** Demographic Characteristics of the Study Population, Study of the Effect of Homeownership on the Prevalence of Chronic Health Conditions, Behavioral Risk Factor Surveillance System, United States, 2020

Characteristic	Unweighted frequency (N = 401,958)^a^	Weighted frequency (N = 260,408,470)	Weighted %^b^
**Age, y**			
18–24	25,652	31,393,114	12.1
25–34	44,382	45,225,090	17.4
35–44	51,971	42,621,840	16.4
45–54	62,033	41,325,747	15.9
55–64	78,089	42,757,627	16.4
≥65	139,831	57,085,052	21.9
**Sex**			
Male	183,931	126,843,330	48.7
Female	218,027	133,565,140	51.3
**Race and ethnicity**			
White, non-Hispanic	303,886	160,824,629	61.8
Black, non-Hispanic	30,390	30,621,521	11.8
Asian, non-Hispanic	10,243	14,622,515	5.6
American Indian/Alaska Native, non-Hispanic	6,954	2,599,155	1.0
Hispanic	36,408	46,273,133	17.8
Other race, non-Hispanic	14,077	5,467,516	2.1
**Marital status**			
Married	207,302	129,628,402	50.3
Divorced	51,939	27,223,100	10.6
Widowed	43,646	17,742,561	6.9
Separated	7,975	6,415,687	2.5
Never married	72,051	63,877,459	24.8
Member of an unmarried couple	15,261	12,889,771	5.0
**Education**			
Did not graduate high school	26,248	32,421,916	12.5
Graduated high school	107,096	71,906,519	27.8
Attended college or technical school	111,387	79,149,739	30.6
Graduated from college or technical school	155,340	75,574,788	29.2
**Employment**			
Employed for wages	165,872	119,253,498	46.8
Self-employed	34,921	22,770,294	8.9
Out of work ≥1 year	7,232	5,978,017	2.4
Out of work <1 year	16,409	14,079,533	5.5
Homemaker	17,080	13,209,384	5.2
Student	10,730	13,267,735	5.2
Retired	117,209	49,944,786	19.6
Unable to work	25,429	16,411,713	6.4
**Annual household income, $**			
<15,000	26,608	19,603,361	9.5
15,000 to <25,000	48,767	31,443,546	15.2
25,000 to <35,000	31,410	19,318,994	9.3
35,000 to <50,000	43,851	26,231,286	12.7
≥50,000	171,265	110,148,555	53.3
**Residential status**			
Own	280,664	171,989,906	66.8
Rent	97,305	69,248,184	26.9
Other arrangements	20,507	16,171,269	6.3

a The sum of column frequencies for certain characteristics may not sum to the total because of missing or excluded data.

b Percentage estimated the population proportion for each level of the variable.

The odds of homeownership increased significantly with advancing age (Table 2). People aged 65 years or older had the highest odds of homeownership (OR = 17.45; 95% CI, 16.21–18.79). Female respondents had a modestly elevated odds of homeownership compared with male respondents (OR = 1.03; 95% CI, 1.00–1.06). Non-Hispanic White people had substantially higher odds of homeownership (OR = 3.34; 95% CI, 3.18–3.52) compared with Hispanic people, while various other racial and ethnic groups exhibited comparatively lower odds. Married people had the highest odds of homeownership (OR = 10.16; 95% CI, 9.71–10.64). Education and income also showed strong positive associations with homeownership. Respondents who graduated from college or technical school had higher odds of homeownership compared with those who did not graduate high school (OR = 4.13; 95% CI, 3.89–4.38), and respondents in higher income brackets had notably higher odds than those who earned less than $15,000 per year. Respondents who had an annual household income of $50,000 or more were 7.8 times more likely to own a home than those earning less than $15,000 annually.

**Table 2:** Association Between Demographic Characteristics and Homeownership, Study of the Effect of Homeownership on the Prevalence of Chronic Health Conditions (N = 401,958), Behavioral Risk Factor Surveillance System, United States, 2020

Characteristic	Unweighted estimate^a^	Weighted estimate^a^
	OR (95% CI)	
**Age, y**		
18–24	1 [Reference]	
25–34	3.11 (3.00–3.23)	2.43 (2.26–2.62)
35–44	7.91 (7.63–8.21)	5.69 (5.29–6.13)
45–54	12.71 (12.25–13.18)	9.11 (8.44–9.83)
55–64	17.63 (17.00–18.29)	12.89 (11.95–13.90)
≥65	25.25 (24.38–26.16)	17.45 (16.21–18.79)
**Sex**		
Male	1 [Reference]	
Female	1.07 (1.10–1.10)	1.03 (1.00–1.06)
**Race and ethnicity**		
White, non-Hispanic	3.69 (3.61−3.77)	3.34 (3.18–3.52)
Black, non-Hispanic	1.11 (1.08−1.14)	1.09 (1.02–1.17)
Asian, non-Hispanic	1.38 (1.32−1.44)	1.69 (1.53–1.88)
American Indian/Alaska Native, non-Hispanic	1.62 (1.54−1.71)	1.53 (1.35–1.74)
Hispanic	1 [Reference]	
Other race, non-Hispanic	1.36 (1.31−1.41)	1.46 (1.34–1.60)
**Marital status**		
Married	12.01 (11.77−12.25)	10.16 (9.71–10.64)
Divorced	2.83 (2.76−2.89)	3.10 (2.94–3.27)
Widowed	6.78 (6.59−6.97)	6.80 (6.34–7.30)
Separated	1.27 (1.22−1.34)	1.34 (1.21–1.48)
Never married	1 [Reference]	
Member of an unmarried couple	1.54 (1.49−1.60)	1.41 (1.30–1.53)
**Education**		
Did not graduate high school	1 [Reference]	
Graduated high school	2.05 (2.00−2.11)	1.82 (1.71–1.94)
Attended college or technical school	2.61 (2.54−2.68)	2.34 (2.20–2.49)
Graduated from college or technical school	4.55 (4.43−4.68)	4.13 (3.89– 4.38)
**Employment**		
Employed for wages	2.52 (2.40−2.64)	2.24 (2.01–2.49)
Self-employed	4.16 (3.95−4.39)	3.15 (2.80–3.55)
Out of work ≥1 year	1 [Reference]	
Out of work <1 year	0.99 (0.93−1.04)	0.97 (0.85–1.10)
Homemaker	3.34 (3.16−3.54)	2.86 (2.51–3.25)
Student	0.31 (0.29−0.33)	0.46 (0.40–0.53)
Retired	7.09 (6.74−7.44)	7.89 (7.05–8.83)
Unable to work	1.04 (0.99−1.1)	1.16 (1.03–1.30)
**Annual household income, $**		
<15,000	1 [Reference]	
15,000 to <25,000	1.88 (1.82−1.94)	1.73 (1.59–1.87)
25,000 to <35,000	2.85 (2.75−2.94)	2.33 (2.13–2.54)
35,000 to <50,000	3.98 (3.85−4.11)	3.54 (3.25–3.85)
≥50,000	8.55 (8.31−8.79)	7.82 (7.25–8.43)

Abbreviation: OR, unadjusted odds ratio.

a Odds ratios were used to examine the association between demographic characteristics and homeownership, estimated through logistic regression. To demonstrate the study design’s effect, both unweighted and weighted estimates were calculated. The weighted odds ratios, along with their corresponding 95% CIs, were adjusted for the study design, providing population-level estimates.

Among people aged 18 to 44 years, homeowners exhibited a significantly higher prevalence of cancer other than skin (1.6% vs 1.2%; *P* = .002), and lower prevalence of asthma (13.9% vs 16.6%; *P* <.001) (Table 3). The prevalence of other conditions was comparable between the 2 groups. Among respondents aged 45 to 64 years, homeowners had a significantly lower prevalence of angina/CHD (3.8% vs 6.0%), ever diabetes (16.3% vs 25.4%), asthma (12.4% vs 17.4%), stroke (2.7% vs 6.2%), and kidney disease (2.6% vs 5.1%) (all *P* <.001). Among people aged 65 to 80 years, homeowners displayed lower prevalence rates of all chronic health conditions than renters, except for cancer other than skin.

**Table 3:** Prevalence of Chronic Health Conditions Among Homeowners and Renters, by Age, Study of the Effect of Homeownership on the Prevalence of Chronic Health Conditions (N = 401,958), Behavioral Risk Factor Surveillance System, United States, 2020

Age group and chronic health condition	Homeowners, %	Renters, %	*P* value^a^
**Aged 18–44 y**			
Angina/coronary heart disease	0.8	0.6	.07
Ever diabetes	5.8	5.8	.98
Cancer other than skin	1.6	1.2	.002
Asthma	13.9	16.6	<.001
Stroke	0.8	0.9	.24
Kidney disease	1.0	1.1	.23
**Aged 45–64 y**			
Angina/coronary heart disease	3.8	6.0	<.001
Ever diabetes	16.3	25.4	<.001
Cancer other than skin	6.6	6.4	.59
Asthma	12.4	17.4	<.001
Stroke	2.7	6.2	<.001
Kidney disease	2.6	5.1	<.001
**Aged 65–80 y**			
Angina/coronary heart disease	11.0	12.4	.02
Ever diabetes	25.0	33.0	<.001
Cancer other than skin	17.0	16.1	.21
Asthma	11.1	15.4	<.001
Stroke	6.9	11.6	<.001
Kidney disease	6.8	9.5	<.001

a *P* values calculated by using Rao-Scott χ^2^ test. Prevalence estimated as the population proportion of the column total stratified by age categories.

After adjusting for age and sex (Model 1), the odds of several chronic health conditions were significantly higher for renters in comparison with homeowners (Table 4). These significant associations remained in Model 2, which was adjusted for age, sex, and race and ethnicity. In Models 3 (adjusted for age, sex, race and ethnicity, education, and marital status) and 4 (adjusted for same variables as Model 3, plus employment and income), the associations remained significant for several conditions. In Model 4, renters had 1.14 (95% CI, 1.03–1.27) higher odds of angina/CHD and 1.15 (95% CI, 1.08–1.23) higher odds of ever diabetes compared with homeowners. Renters also had 1.18 (95% CI, 1.11–1.22) higher odds of asthma and 1.34 (95% CI, 1.19–1.52) higher odds of stroke in comparison to homeowners. Similarly, renters had 1.38 higher odds of kidney disease compared with homeowners (95% CI, 1.22–1.56).

**Table 4:** Association Between Homeownership and Prevalence of Chronic Health Conditions (N = 401,958)^a^, Behavioral Risk Factor Surveillance System, United States, 2020

Chronic health condition	Model 1: adjusted for age and sex	Model 2: adjusted for age, sex, and race/ethnicity	Model 3: adjusted for age, sex, race/ethnicity, education, marital status	Model 4: adjusted for age, sex, race/ethnicity, education, marital status, employment	Model 5: adjusted for age, sex, race/ethnicity, education, marital status, employment, income
	AOR (95% CI)				
Angina/coronary heart disease	1.33 (1.22–1.44)	1.39 (1.27–1.52)	1.31 (1.18–1.44)	1.14 (1.03–1.27)	1.05 (0.95–1.16)
Ever diabetes	1.49 (1.41–1.57)	1.27 (1.20–1.35)	1.24 (1.16–1.33)	1.15 (1.08–1.23)	1.05 (0.98–1.13)
Cancer other than skin	0.97 (0.90–1.04)	1.06 (0.99–1.14)	1.11 (1.02–1.21)	1.03 (0.95–1.13)	1.04 (0.96–1.14)
Asthma	1.28 (1.22–1.35)	1.29 (1.23–1.36)	1.24 (1.17–1.31)	1.18 (1.11–1.22)	1.13 (1.07–1.21)
Stroke	2.00 (1.82–2.19)	1.89 (1.71–2.09)	1.58 (1.40–1.78)	1.34 (1.19–1.52)	1.18 (1.04–1.33)
Kidney disease	1.68 (1.53–1.85)	1.59 (1.44–1.77)	1.55 (1.38–1.75)	1.38 (1.22–1.56)	1.27 (1.12–1.43)

Abbreviation: AOR, adjusted odds ratio.

a Logistic regression models were adjusted for study design. Reference group was homeowners. Odds ratios examined association between prevalence of chronic health condition and homeownership status (ie, living in rental house vs living in own house). For adjusted models, certain categories of marital status, education, and employment were collapsed. Marital status: categories 1 and 6 (“married” and “member of an unmarried couple”) and categories 2, 3, and 4 (“divorced,” “widowed,” and “separated”) were combined. Education: categories 1 and 2 (“did not graduate high school” and “graduated high school”) and categories 3 and 4 (“attended college or technical school” and “graduated from college or technical school”) were combined. Employment: categories 1, 2, and 5 (“employed for wages,” “self-employed” and “homemaker”) and categories 3, 4, and 8 (“out of work ≥1 year,” “out of work for <1 year,” and “unable to work”) were combined.

## Discussion

We found distinct associations between demographic characteristics and homeownership status, with age, race and ethnicity, education, and income significantly influencing ownership. The prevalence of chronic health conditions varied significantly among different age groups and homeownership status. Notably, homeowners displayed a lower prevalence of self-reported angina/CHD, ever diabetes, asthma, stroke, and kidney disease, particularly among people aged 45 to 64 years.

The observed associations between homeownership and chronic health conditions highlight the importance of housing as a social determinant of health. These findings are consistent with the existing literature that emphasizes the multifaceted impact of housing and housing type on health outcomes (15–17). Our study findings align with prior research indicating that housing and homeownership contribute to improved economic security, generational wealth, and social stability (4,5). Homeownership, as a means of wealth accumulation, can provide people and families with resources that positively influence health and well-being (3,5,11,17). The significant associations between homeownership and chronic health conditions remained robust even after adjusting for various demographic factors. This suggests that homeownership's influence on health outcomes transcends individual characteristics, highlighting its unique role in shaping health disparities. The observed effect could be attributed to factors such as differences in housing quality, neighborhood environments, and access to health care services based on homeownership status (3,10,11). Homeowners often experience greater housing stability, which has been linked to better health outcomes (3,8,18). Moreover, homeowners may have more control over their living conditions, leading to improved internal housing conditions that positively impact health (18,19).

The significant impact of homeownership on angina/CHD and diabetes diminished and became less pronounced on other chronic health conditions when income was introduced into the logistic regression model (ie, Model 5). This phenomenon may be attributed to over-adjustment resulting from including income as a covariate alongside homeownership in the model. Our assessment of collinearity between homeownership and income using a χ^2^ test showed a strong correlation. Additionally, we found a consistent dose–response relationship observed for homeownership within each income category in the logistic regression analysis. As expected, the results demonstrated that as income groups increased from 1 (ie, less than $15,000 annual income) to 5 (ie, $50,000 or more), the likelihood of homeownership significantly increased, with people in income group 5 being 7.8 times more likely to own a home than those in income group 1, with a consistent dose response. These findings imply a robust positive association between income level and homeownership, which may have influenced the significance of the adjusted models (Model 5). The significant impact of homeownership on angina/coronary heart disease and diabetes decreased when additional variables were introduced into the logistic regression equation (Model 5), which may be attributed to the multicollinearity between sociodemographic variables.

Although not significant in the age- and sex-adjusted logistic regression model, renters initially displayed lower odds of cancer other than skin. However, with the inclusion of race, education, and marital status, the odds ratio shifted from protective to being at risk, indicating increased odds of cancer other than skin among renters. This shift in odds suggests socioeconomic influences. The reversal in more comprehensive models may indicate the role of social determinants like education and marital status, highlighting potential health disparities tied to housing and socioeconomic conditions. The attenuation of odds ratios with employment and income inclusion underscores the nuanced relationship between homeownership, demographics, and cancer prevalence.

The observed association between age and homeownership is plausibly explained by the accumulation of generational wealth, where the odds of ownership significantly increase with age. This alignment is grounded in the concept that older people, having had more time to accumulate wealth, are better positioned for homeownership. Furthermore, it acknowledges the potential for intergenerational wealth transfer, contributing to a positive cycle and suggesting that policies supporting homeownership may yield long-term positive effects on health and well-being. Homeownership stands as a robust proxy for a range of sociodemographic and income variables, offering easy and less biased measurement. People are more inclined to respond, resulting in fewer missing data. Integrating homeownership into SDOH screening tools in health care settings empowers health care providers to customize care plans based on individual needs. This approach facilitates the identification of vulnerable populations and enables the precise implementation of targeted interventions and preventive measures. Moreover, homeownership surpasses mere housing in significance, illustrating the continuity and longevity of health benefits associated with stable and sustainable housing. This underscores the importance of exploring how promoting homeownership through interventions can substantially enhance community health — a critical frontier in public health research. To maximize its potential as a driver of community well-being, a comprehensive investigation into the link between homeownership and health outcomes is essential.

While this study contributes valuable insights into the relationship between homeownership and chronic health conditions, it has limitations. The study relied on self-reported data, which might introduce recall bias or misclassification of chronic health conditions. Additionally, the cross-sectional nature of the study limits causal inference, and unmeasured confounders might influence the observed associations.

In conclusion, this study provides evidence of a significant association between homeownership and the prevalence of several chronic health conditions in the US. The findings emphasize the need for comprehensive housing-related interventions as part of public health initiatives aimed at enhancing overall health outcomes. Addressing disparities in homeownership could have far-reaching effects on health equity, economic security, and social well-being. Future research could delve deeper into the mechanisms underlying the homeownership–health relationship and evaluate the effectiveness of housing-focused interventions on the prevention and health promotion of chronic health conditions prevention and health promotion.
